# Functional Polymorphisms in the p53 Pathway Genes on the Genetic Susceptibility to Zika Virus Teratogenesis

**DOI:** 10.3389/fcimb.2021.641413

**Published:** 2021-07-07

**Authors:** Julia A. Gomes, Eduarda Sgarioni, Igor A. Vieira, Lucas R. Fraga, Patrícia Ashton-Prolla, Ana Cláudia P. Terças-Tretell, Juliana H. da Silva, Bethânia F.R. Ribeiro, Marcial F. Galera, Thalita M. de Oliveira, Maria Denise F. Carvalho de Andrade, Isabella F. Carvalho, Lavínia Schuler-Faccini, Fernanda S. L. Vianna

**Affiliations:** ^1^ Programa de Pós-Graduação em Genética e Biologia Molecular (PPGBM), Universidade Federal do Rio Grande do Sul (UFRGS), Porto Alegre, Brazil; ^2^ Sistema Nacional de Informação sobre Agentes Teratogênicos (SIAT), Serviço de Genética Médica, Hospital de Clínicas de Porto Alegre (HCPA), Porto Alegre, Brazil; ^3^ Instituto Nacional de Genética Médica Populacional (INAGEMP), Porto Alegre, Brazil; ^4^ Laboratório de Medicina Genômica (LMG), Centro de Pesquisa Experimental (CPE), Hospital de Clínicas de Porto Alegre (HCPA), Porto Alegre, Brazil; ^5^ Departamento de Ciências Morfológicas, Instituto de Ciências Básicas da Saúde, Universidade Federal do Rio Grande do Sul, Porto Alegre, Brazil; ^6^ Departamento de Enfermagem, Universidade do Estado de Mato Grosso (UNEMAT), Tangará da Serra, Brazil; ^7^ Secretaria Municipal de Saúde de Tangará da Serra, Tangará da Serra, Brazil; ^8^ Fundação Hospital das Clínicas do Acre (FUNDACRE), Rio Branco, Brazil; ^9^ Departamento de Pediatria, Faculdade de Medicina, Universidade Federal de Mato Grosso (UFMT), Cuiabá, Brazil; ^10^ Hospital Universitário Júlio Müller (HUJM), Universidade Federal de Mato Grosso (UFMT), Cuiabá, Brazil; ^11^ Curso de Medicina, Universidade Estadual do Ceará (UECE), Fortaleza, Brazil; ^12^ Curso de Odontologia, Centro Universitário Christus (UNICHRISTUS), Fortaleza, Brazil

**Keywords:** congenital abnormalities, Zika virus infection, teratogens, genetic polymorphism, disease susceptibility, risk factors, apoptosis, lissencephaly

## Abstract

Congenital Zika Syndrome (CZS) occurs in up to 42% of individuals exposed to ZIKV prenatally. Deregulation in gene expression and protein levels of components of the p53 signaling pathway, such as p53 and MDM2, due to ZIKV infection has been reported. Here, we evaluate functional polymorphisms in genes of the p53 signaling pathway as risk factors to CZS. Forty children born with CZS and forty-eight children exposed to ZIKV, but born without congenital anomalies were included in this study. Gestational and sociodemographic information as well as the genotypic and allelic frequencies of functional polymorphisms in *TP53, MDM2, MIR605* and *LIF* genes were compared between the two groups. We found children with CZS exposed predominantly in the first trimester and controls in the third trimester (p<0.001). Moreover, children with CZS were predominantly from families with a lower socioeconomic level (p=0.008). We did not find a statistically significant association between the investigated polymorphisms and development of CZS; however, by comparing individuals with CZS and lissencephaly or without lissencephaly, we found a significative difference in the allelic frequencies of the *TP53* rs1042522, which is associated with a more potent p53-induced apoptosis (p=0.007). Our findings suggest that the *TP53* rs1042522 polymorphism should be better investigate as a genetic risk factor for the development of lissencephaly in children with CZS.

## Introduction

Zika virus (ZIKV) is a human teratogen that infects neural cells of children exposed during pregnancy and causes a spectrum of multiple congenital anomalies named Congenital Zika Syndrome (CZS), which includes microcephaly, brain calcifications, lissencephaly, ventriculomegaly, ocular alterations, among others (del Campo et al., 2017). It has been reported that up to 42% of all individuals exposed to ZIKV during pregnancy indeed develop the CZS (Nithiyanantham and Badawi, 2019).

Recently, *in silico* and *in vitro* studies have shown that the p53 protein, encoded by the *TP53* gene, is differentially expressed in human neuroprogenitor cells (hNPCs) during ZIKV infection (El Ghouzzi et al., 2016; Zhang et al., 2016). Importantly, the increase in the *TP53* expression under this condition induce cell cycle arrest and death of developing neurons, and it has been proposed as a possible mechanism for ZIKV teratogenesis (El Ghouzzi et al., 2016; Bhagat et al., 2018). Through a systems biology approach, it was identified that p53 is the central protein of a genetic regulatory network of proteins associated with the ZIKV infection and those associated with microcephaly occurrence (Teng et al., 2017). In addition, it was demonstrated that increased p53 activity leads to an increased death rate of neural cells due to the binding of the ZIKV capsid protein to MDM2 protein, decreasing its activity as one of the main p53 negative regulators (Teng et al., 2017).

In a context of wild-type *TP53* sequence, constitutive p53 protein levels and activity can be modulated directly or indirectly by several regulator proteins and specific microRNAs (miRNAs) (Liu and Chen, 2006; Hermeking, 2012). LIF, a cytokine encoded by the *LIF* gene, is a p53 regulator that can both indirectly repress p53 functions in human cells through the induction of MDM2, or it can be up-regulated by p53 itself (Liu et al., 2015). Like p53, LIF protein appears to be increased in brain cells infected by ZIKV (Bayless et al., 2016). Recently, the role of miRNAs has been described in ZIKV infection, and dysregulation of nervous system development pathways mediated by several miRNAs was demonstrated (Bhagat et al., 2018). The miR-605 is an miRNA that acts as an indirect modulator of *TP53* gene expression through the negative regulation that it exerts targeting *MDM2* transcripts (Xiao et al., 2011).

Based on previous evidence of differential susceptibility to ZIKV teratogenesis in humans, as well as possible involvement of the p53 signaling pathway in this outcome, we investigated the role of functional single nucleotide variants (SNVs) in genes of the p53 signaling pathway as potential susceptibility factors to CZS in a sample of Brazilian children exposed to ZIKV during pregnancy. In addition, we evaluated the impact of ZIKV infection on the expression of genes of this pathway in neuroprogenitor cells, the main targets of ZIKV in the developing brain.

## Materials and Methods

### Ethical Issues

This study was carried out following the rules of the Declaration of Helsinki and approved by the Ethics and Research Committee of the Hospital de Clínicas de Porto Alegre, institution responsible for this study (n° 170619 – CAAE 78735817910015327), and by all participating institutions. All legal guardians of individuals recruited for this study gave their informed consent for inclusion before they participated in the study.

### Sample

This case-control study was conducted with 88 children exposed to the ZIKV infection during pregnancy. Forty children were born with CZS and 48 were born without congenital anomalies. Recruitment for the study required evidence of ZIKV exposure, defined as a positive result in the RT-PCR test to detect viral RNA or typical symptoms of infection (e.g. rash, fever and joint pain) during any time of the pregnancy. Patients in the CZS group (n=40) were recruited from reports of microcephaly in five Brazilian research and/or assistance centers: North region (Fundação Hospital das Clínicas do Acre, n=4), Northeast (Universidade Estadual do Ceará, n=21), Midwest (Universidade do Estado de Mato Grosso, n=2 and Hospital Universitário Júlio Müller, n=12) and South (Hospital de Clínicas de Porto Alegre, n=1). Control group was recruited in the same centers from the North region (n=1), Midwest (n=46, from a cohort of women that gave birth in 2016, in the city of Tangará da Serra) and South (n=1) of the country.

Sociodemographic and pregnancy characteristics were obtained from questionnaires answered by mothers during medical consultation. Clinical data (dysmorphological features and, when available, the results of the neuroimaging and ophthalmological exams) were obtained from chart review and direct consultation and interviews done by physicians.

### Genetic Analysis

Blood or saliva samples were collected from participants and the DNA extraction was performed using FlexiGene DNA (Qiagen^®^) or Oragene (DNA Genotek) kits. Functional single nucleotide variants (SNVs) in *TP53, MDM2, MIR605* and *LIF* were selected based on their previous description as important modulators of the p53 pathway genes, either through control of expression levels or function of proteins (Dumont et al., 2003; Bond et al., 2004; Pim and Banks, 2004; Id Said and Malkin, 2015; Moudi et al., 2020). Genotyping was performed using the TaqMan^®^ Genotyping Assay method in a Step One Plus™ Real-Time PCR System (Applied Biosystems, Carlsbad, USA). The reference SNV numbers included in our analyses, the commercial assay codes of TaqMan^®^ probes employed, and the allelic discrimination of each probe were as follows: *TP53* rs1042522 [C/G] (c:2403545_10), *MDM2* rs2279744 [T/G] (c:15968533_20), *MIR605* rs2043556 [C/T] (c:11737438_10) and *LIF* rs929271 [G/T] (c:7545904_10). The allelic and genotypic frequencies were determined based on the genotyping results of each group. For allelic frequencies, the number of times an allele was identified in each group (case or control) was divided by the total number of alleles present in each group for each polymorphism (e.g. for 40 individuals in the case group, we had a total of 80 alleles for each polymorphism). The allelic frequencies obtained for each group were compared with the frequencies reported in the Brazilian population (based on the AbraOM database - http://abraom.ib.usp.br/) and in populations all over the world (based on the Genome Aggregation Database (gnomAD) and 1000 Genomes Project data obtained from Ensembl database - https://www.ensembl.org/index.html). Since the Brazilian population is highly admixed and the individuals in this sample come from different regions of Brazil, we decided not to compare the allelic frequencies with a specific population (e.g. European or Latin American populations), but with data from the world population.

### Statistical Analyses

Descriptive analysis of the congenital anomalies was performed in the CZS group. Quantitative variables were tested through the Shapiro-Wilk test to verify their normality and from this, Student’s t test or Mann–Whitney U test were applied to compared the groups. Hardy-Weinberg equilibrium was tested for all SNVs. Categorical variables were compared between groups by Chi-squared Test or Fisher’s Exact Test. A p-value <0.05 was considered statistically significant. The SPSS^®^ v.20 software (SPSS Inc., Chicago, USA) was used for data handling and for all statistical analyses.

### Gene Expression Analyses

In order to better understand the effect of ZIKV infection on the gene expression of *TP53*, *MDM2, MIR605* and *LIF*, the expression data of human neuroprogenitor cells exposed and not exposed to ZIKV were evaluated. For this evaluation, the raw data from the study GSE129180 (Liu et al., 2019), available in the Gene Expression Omnibus (GEO) database, were obtained (https://www.ncbi.nlm.nih.gov/geo/). **Supplementary Table 1** presents the characteristics of this study.

Raw data from RNA-seq analysis were downloaded from the European Nucleotide Archive (ENA) database (https://www.ebi.ac.uk/ena/browser/home) in.fastq format and re-processed. The quality control of the samples was evaluated by FastQC tool v.0.11.7 (Andrews, 2010) and they were processed in the Galaxy platform (Afgan et al., 2018). Reads were aligned against the human genome reference sequence (GRCh38) using the short-read aligner Bowtie v1.2.0 or Bowtie2 v2.3.4.3 (Langmead, 2010; Langmead and Salzberg, 2012). The read count was performed with HTseq-count v.0.9.1 (Anders et al., 2015) to estimate the abundance of transcripts expression. The generated outputs were downloaded to be evaluated.

Differential gene expression analysis was performed in R v.3.6.2 applying robust multiaverage (RMA) normalization and using limma package (Ritchie et al., 2015). ZIKV infected cells were compared to non-infected cells. Genes with | log2 (fold change) | > 1.5 or <1.5 and false discovery rate (FDR) ​​<0.05 were considered to be statistically significant and differentially expressed.

## Results

The clinical and sociodemographic description of the sample used in this study was previously performed in another study by our group (Gomes et al., 2021). However, we summarize here in **Table 1** the clinical, gestational, and sociodemographic characteristics of both study groups. Mothers of children with CZS had ZIKV infection predominantly in the first trimester of pregnancy (80%) while mothers of children without congenital anomalies reported infection predominantly in the third trimester (44%) (p<0.001). Mothers of children with CZS exhibited a lower educational level (p<0.001) and lower monthly family incomes compared to mothers of children without congenital malformations (p=0.008). Children without CZS had higher weight, height and cephalic perimeter measures compared to with CZS.

**Table 1:** Evaluation of clinical, gestational, and sociodemographic characteristics in the case (CZS) and control (without CZS) groups.

Variables	Cases^a^ (n=40)	Controls (n=48)	*p-*value^b^
Sex (n, %)			
*Male*	23 (57%)	26 (54%)	0.754
*Female*	17 (43%)	22 (46%)	
Ethnicity (n, %)			
*Black *	31 (77%)	31 (65%)	0.242
*White*	9 (23%)	17 (35%)	
Weight (kg)	2.5 (2.2 - 2.9)	3.2 (2.8 - 3.5)	<0.001*
Height (cm)	45.0 (44.0 - 48.0)	49.0 (47.0 - 50.0)	0.001*
Cephalic perimeter (cm)	29.0 (27.3 - 31.0)	35.0 (34.0 - 36.0)	<0.001*
Gestational age at birth (weeks)	38.0 (37.0 - 39.0)	38.0 (37.0 - 38.7)	0.522
Types of delivery (n, %)			
*Vaginal delivery*	16/30 (53%)	5 (10%)	<0.001*
*Cesarean section*	14/30 (47%)	43 (90%)	
Mother’s age (years)	28.0 (22.5 - 35.5)	29.5 (22.0 - 33.0)	0.824
Father’s age (years)	28.0 (24.0 - 37.8)	31.0 (26.0 - 35.0)	0.693
Trimester of ZIKV infection (n, %)			
*1st*	24/30 (80%)	13 (27%)	<0.001*
*2nd*	4/30 (13%)	14 (29%)	
*3rd*	2/30 (7%)	21 (44%)	
Exposure during pregnancy (n, %)			
*Alcohol*	3/35 (9%)	14 (29%)	0.028*
*Smoke*	0/35	1 (2%)	1.000
*Recreational drugs*	0/35	1 (2%)	1.000
Maternal yellow fever vaccine (n, %)	18/24 (75%)	35 (73%)	1.000
Maternal educational level (n, %)			
*Elementary school*	13/38 (34%)	0	<0.001*
*High school*	13/38 (34%)	2 (4%)	
*Incomplete or complete higher education*	12/38 (32%)	46 (96%)	
Monthly family income (n, %)			
*Less than 3 minimum wages*	23/27 (85%)	12/25 (46%)	0.008*
*Between 3 and 9 minimum wages*	4/27 (15%)	12/25 (46%)	
*More than 9 minimum wages*	0	2/25 (8%)	

^a^In the case group, some informations were not available or were not answered for all mothers; ^b^Quantitative variables were compared between the groups through the Student’s t test or Mann–Whitney U test and categorical variables through the Chi-squared test or Fisher’s exact test; Quantitative variables are presented as median and quartiles; *Statistically significant.

**Figure 1:**
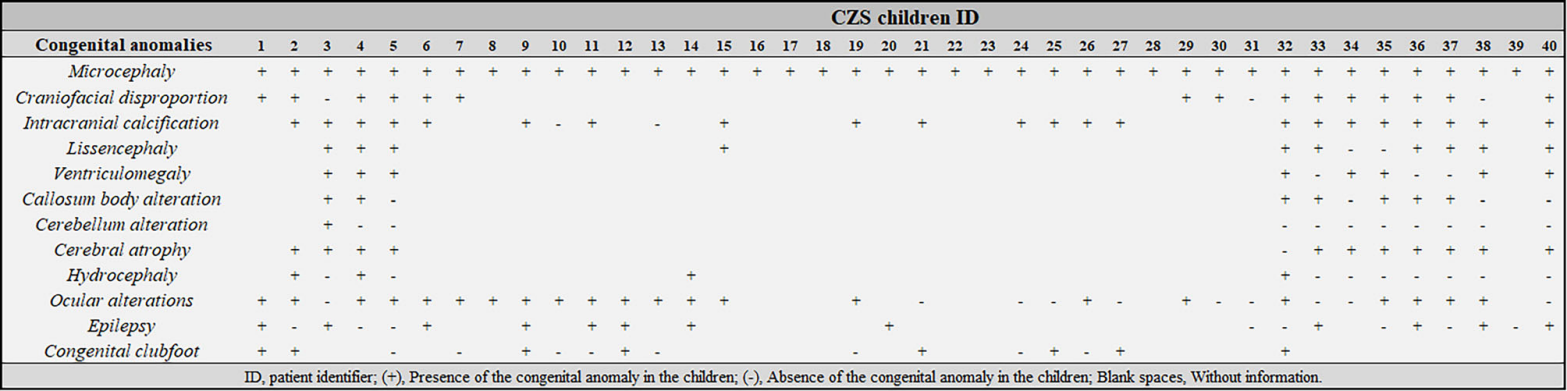
Clinical characterization of children with Congenital Zika Syndrome (CZS) included in this study. ID, patient identifier; (+), Presence of the congenital anomaly in the children; (-), Absence of the congenital anomaly in the children; Blank spaces, Without information.

Congenital anomalies observed in children with CZS also were previously described in the aforementioned study (Gomes et al., 2021), but they are summarized in **Figure 1**. Detailed dysmorphologic, ocular and imaging exams were not carried out on all individuals with CZS, since some research and assistance centers had limited resources to perform such tests. The most prevalent clinical findings were microcephaly (n=40, 100%) brain calcifications (n​​=2/24, 92%), cerebral atrophy (n=11/12, 92%), lissencephaly (n=10/12, 83%), craniofacial disproportion (n=15/18, 83%), ventriculomegaly (n=8/11, 73%), and ocular alterations (n=22/32, 69%).

**Figure 2:**
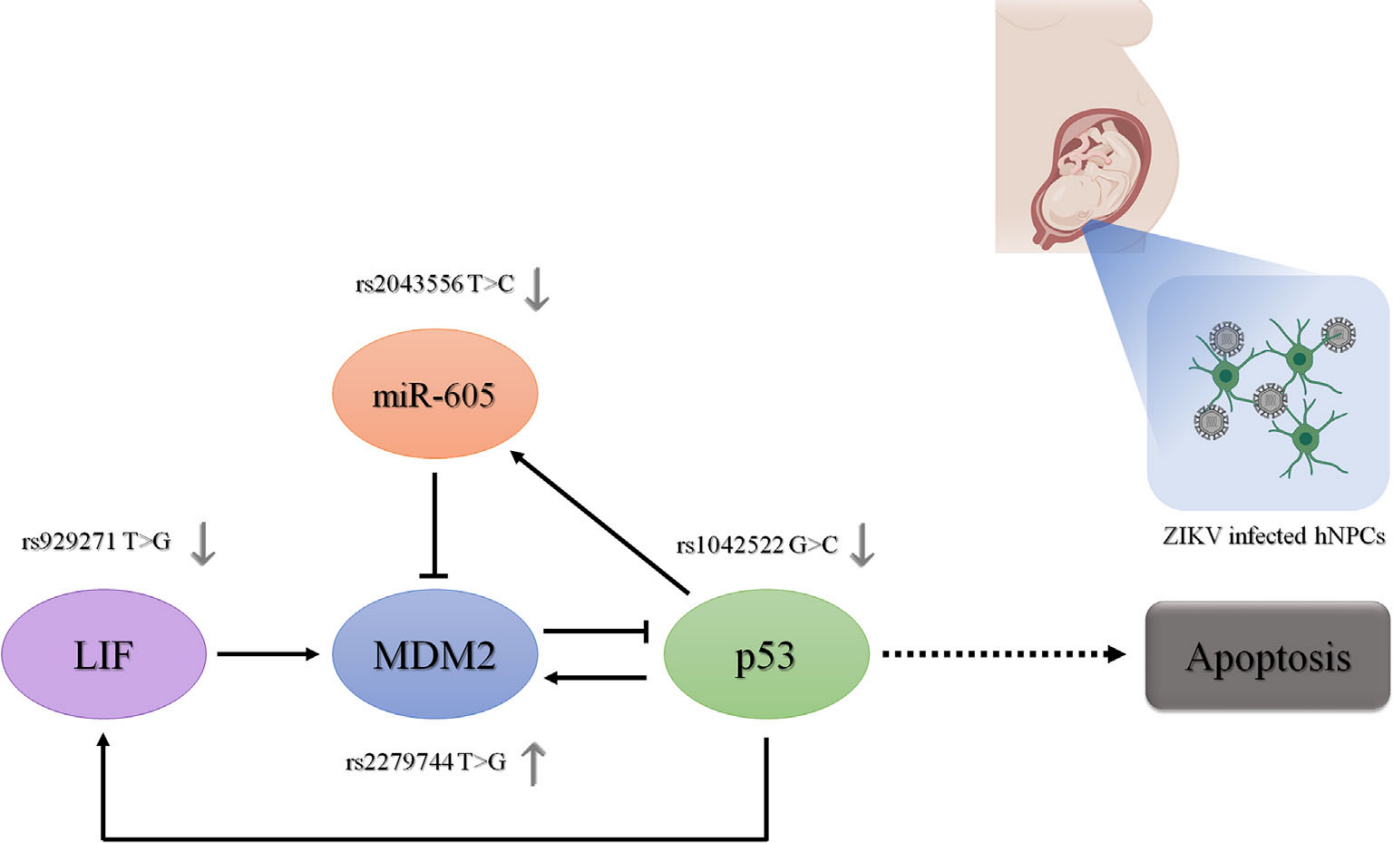
Role of the investigated genes and their functional polymorphisms in the p53 signaling pathway. The regulatory role of the proteins encoded by these genes in the expression of the other genes in the p53 signaling pathway is represented by dark arrows. The regulatory role of the polymorphisms in their gene expression is represented by gray arrows. Ultimately, the effect of these genetic variants on p53 expression levels and activity in the context of human neural cells infected by ZIKV is represented. hNPCs, human neuroprogenitor cells. Created in BioRender.com.

**Figure 2** provides a summary of the proposed relationships among genes and SNVs studied here and their interplay in regulating the p53 pathway. Regarding the allelic and genotypic frequencies of the SNVs selected in *TP53, MDM2, MIR605* and *LIF*, they were not statistically different between CZS and control groups (**Supplementary Table 2**). Comparing the allelic frequencies obtained in the case and control groups with the frequencies reported in the Brazilian and worldwide population, we observed that they were quite similar (**Supplementary Table 2**), but it is important to note that the Brazilian population is highly admixed (Kehdy et al., 2015), and the allelic and genotypic frequencies of the our population are not always similar to other specific populations.

Allelic frequencies were also compared, within the group of children with CZS, between those who presented or not specific brain malformations, such as intracranial calcification, lissencephaly, cerebral atrophy, hydrocephaly and ocular alterations (information available for more than 12 individuals) (**Supplementary Table 3**). A significative difference in the allelic frequencies of the SNV *TP53* rs1042522 was found between individuals with CZS and lissencephaly or without lissencephaly. Interestingly, we detected the G allele with a frequency lower than expected in individuals with lissencephaly and much higher than expected in individuals without lissencephaly (p=0.007) (**Table 2**). Genotypic frequencies were also different between groups, but this difference was tangential to the significance.

**Table 2:** Allelic and genotypic frequencies of *TP53* rs1042522 polymorphism in children with CZS and lissencephaly or with CZS and without lissencephaly.

Gene	Polymorphism	Allele/Genotype	CZS and lissencephaly (n=10)^a^	CZS without lissencephaly (n=2)^a^	*P value*
***TP53* (n, %)**	**rs1042522 (missense)**	C	16 (80%)	0	0.007*
		G	4 (20%)	4 (100%)	
		CC	7 (70%)	0	0.061
		GC	2 (20%)	0	
		GG	1 (10%)	2 (100%)	

^a^Clinical information was not available for all individuals with CZS. *Statistically significant.

Gene expression analysis showed an increased expression of *LIF* in human neuroprogenitor cells three days after the infection by the Asian strain of ZIKV, but this association lost its significance after correcting the p-value (logFC = 1.41, p = 0.001, FDR = 0.44). The other genes did not have their expression disturbed by the ZIKV infection (**Supplementary Table 4**).

## Discussion

The ability of ZIKV to impair the central nervous system development is still poorly understood. Although it is known that only up to 42% of individuals exposed to ZIKV develop CZS, genetic components related to this susceptibility have not yet been recognized (Nithiyanantham and Badawi, 2019). In this study we assessed the role of selected SNVs in p53 pathway genes as potential susceptibility factors to CZS in a sample of Brazilian children exposed to ZIKV during pregnancy. Besides, we evaluated how ZIKV infection deregulates the expression of the same genes in neuroprogenitor cells.

The p53 signaling pathway was chosen for investigation in this study because it has been shown to be affected by ZIKV during infection (El Ghouzzi et al., 2016; Tang et al., 2016; Teng et al., 2017). Similarly, the dysregulation of miRNAs in human neuronal cells during ZIKV infection has also been reported by several studies (Bhagat et al., 2018). Considering these findings and the well-documented miRNA network acting in the direct and indirect regulation of the p53 pathway, we also evaluated in this work an SNV in a miRNA that has been shown to positively regulate p53 activity (Xiao et al., 2011; Hermeking, 2012).

Although we did not find an association between the studied SNVs and susceptibility to CZS, we found a lower frequency of individuals with CZS and lissencephaly carrying the G-allele of the *TP53* rs1042522 while children without lissencephaly had this allele in homozygosis. Lissencephaly comprises a group of disorders characterized by an abnormally smooth surface of the cerebral cortex due an abnormal neuronal migration, where genes related to the neural migration process are usually mutated (Mochida, 2009). The G-allele of the *TP53* rs1042522 has been associated with higher p53 activity and more potent capacity to induce apoptosis (Dumont et al., 2003). During ZIKV infection, p53 expression has already been shown to be upregulated in an attempt to decrease viral proliferation and eliminate the virus (El Ghouzzi et al., 2016; Tang et al., 2016). In this context, individuals carrying the G allele could have an advantage in viral elimination, with more efficient apoptosis, while those carrying the C allele would allow the virus to spread on a larger scale, impairing the differentiation and activity of a greater number of neural cells. Despite being biologically consistent with the development of an altered phenotype, such as lissencephaly, this result should be interpreted with caution, since this association was identified in a small subset of patients. Therefore, additional case-control studies with a larger sample size and extensive clinical characterization are required to confirm this finding.

The ability of ZIKV infection to affect the expression of p53 pathway genes (El Ghouzzi et al., 2016; Zhang et al., 2016) was one of the main reasons that led to the development of this study. Thus, to evaluate how ZIKV affected the expression of *TP53, MDM2, MIR605* and *LIF* in hNPCs, we performed an analysis of differential gene expression in exposed cells compared to unexposed cells. The study used for such analysis (Liu et al., 2019 – GSE129180) was different from those cited as a reference previously (El Ghouzzi et al., 2016 – GSE78711; Zhang et al., 2016 – GSE80434). In this analyze, we found only the upregulation of *LIF* due to ZIKV infection, not corroborating the previously reported findings on *TP53* and *MDM2* (El Ghouzzi et al., 2016; Zhang et al., 2016) and not identifying an alteration in the *MIR605* expression. However, it is important to mention that although the cell type evaluated by these three studies was the same (hNPCs), the differences in the lineage of the ZIKV(Asian or African) and the time of exposure to ZIKV have a strong influence in which genes are affected and how significant is the altered expression. In this sense, we saw that in the study by El Ghouzzi and colleagues (2016) the analysis of the differential gene expression occurred 72 hours after infection by the Asian strain of ZIKV, while in the study by Zhang et al. and colleagues (2016) the evaluation was 64 hours after infection by the Asian strain, and in the study by Liu et al. (2019) the analysis occurred 3 days (72h) and 6 days after infection by the Asian lineage, which means that no study has used the same methodological approaches and, consequently, different results can be expected.

Regarding the gestational and socio-environmental features, the first trimester of ZIKV infection has been associated with the development of more severe congenital anomalies and abortions, while the socioeconomic level, closely related to nutritional and health conditions, could be discussed as a possible risk factor for ZIKV teratogenesis (França et al., 2016; Barbeito-Andrés et al., 2020). In this sense, our study corroborated the previous findings on increased risk to CZS due to ZIKV exposure during the first trimester (França et al., 2016). In addition, our findings suggest that socioeconomic level may be an important risk factor, which deserves to be further explored in the context of ZIKV teratogenesis.

Lastly, this study has some limitations that must be considered in the interpretation of the results, such as the sample size, which affects our power to identify strong associations or to perform more robust statistical analyses. The size of our complete sample is small (n = 88) and when subgroup comparisons are made, it becomes even smaller. Thus, considering our sample size and based on studies that have already performed comparisons of allelic frequencies of polymorphisms in samples of individuals exposed to ZIKV during pregnancy (Santos et al., 2019; Gomes et al., 2021), the power of the present study to identify significant associations was approximately 40%. In this sense, we highlight the importance of future studies being carried out on larger samples, making it possible to confirm the associations found by this study, as well as carrying out more robust statistical analyzes, such as the allele size effect. Currently, it is especially difficult to increase the sample size, since fortunately the ZIKV outbreak in Brazil has decreased since 2017. However, it is important to emphasize that this is one of the most largest cohort of children exposed to ZIKV during pregnancy used in the context of studies on genetic susceptibility to ZIKV teratogenesis (Caires-Júnior et al., 2018; Candelo et al., 2019; Santos et al., 2019; Aguiar et al., 2020; Gomes et al., 2021). The lack of detailed data on gestational history and clinical description for some patients is also a limitation of this study, motivated by the regional variations in available resources during medical consultation. The different origin of the CZS and control groups may also constitute a confounding factor in the study, because the number of cases from each Brazilian region was not matched with the same number of controls for the same region, and, considering that the Brazilian population is a very admixed, it is reasonable to speculate that differential genetic variability between groups might have been observed for that reason and may not strictly related to the development of CZS.

In conclusion, although some studies have described that p53 pathway genes seems to be affected by ZIKV infection and they need attention given to their essential functions in embryonic and fetal development and apoptosis, in the present study we did not find any association between selected variants in genes of this pathway and an increased risk to ZIKV teratogenesis. Importantly, we identified a possible association between *TP53* rs1042522 and the occurrence of lissencephaly in CZS patients, which should be further explored in additional studies. We emphasize that, although biological pathways involved in the teratogenesis of ZIKV have been reported in recent years, additional efforts are still needed to elucidate the main genetic factors implicated in the susceptibility to CZS. Additional case-control and functional studies focusing on both SNVs located in p53 pathway genes and in specific miRNA genes regulating ZIKV-mediated teratogenesis should be performed on individuals exposed to ZIKV in order to better understand their role in the CZS development.

## Data Availability Statement

The raw data supporting the conclusions of this article will be made available by the authors, without undue reservation.

## Ethics Statement

This study involved human participants and, therefore, was reviewed and approved by Ethics and Research Committee of the Hospital de Clínicas de Porto Alegre - n° 170619 – CAAE 78735817910015327. Written informed consent to participate in this study was provided by the participants’ legal guardian/next of kin.

## Author Contributions

JAG, LS-F, and FSLV: conceptualization of the study. JAG, ES, IAV, ACPTT, JHS, BFRR, MFG, TMO, MDFCA, and IFC: acquisition, analysis, and interpretation of the data. JAG: writing—original draft preparation. JAG, LRF, PA-P, LS-F, and FSLV: writing—review and editing. JAG, LS-F, and FSLV: supervision, project administration and funding acquisition. All authors contributed to the article and approved the submitted version.

## Funding

This work was supported by the Instituto Nacional de Genética Médica Populacional (INAGEMP) [grant number CNPq 573993/2008-4, FAPERGS 17/2551.0000521-0], Fundo de Incentivo a Pesquisa e Eventos (FIPE) of the Hospital de Clínicas de Porto Alegre (HCPA) [grant numbers 2017-0619, 2019-0295], Conselho Nacional de Desenvolvimento Científico e Tecnológico [grant numbers 424362/2018-0 and Chamada CNPq/CAPES/MS-14/2016]. FSLV is recipient of a CNPq scholarship grant [grant number CNPq 312993/2017-0]. PA-P is recipient of a CNPq scholarship grant [grant number CNPq 307826/2017-1].

## Conflict of Interest

The authors declare that the research was conducted in the absence of any commercial or financial relationships that could be construed as a potential conflict of interest.

## References

[B1] Afgan E. Baker D. Batut B. van den Beek M. Bouvier D. Cech M.. (2018). The Galaxy Platform for Accessible, Reproducible and Collaborative Biomedical Analyses: 2018 Update. Nucleic Acids Res. 46, W537–W544. 10.1093/nar/gky379 29790989 PMC6030816

[B2] Aguiar R. S. Pohl F. Morais G. L. Nogueira F. C. S. Carvalho J. B. Guida L.. (2020). Molecular Alterations in the Extracellular Matrix in the Brains of Newborns With Congenital Zika Syndrome. Sci. Signal. 13, eaay6736. 10.1126/scisignal.aay6736 32518143

[B3] Anders S. Pyl P. T. Huber W. (2015). HTSeq–a Python Framework to Work With High-Throughput Sequencing Data. Bioinformatics 31, 166–169. 10.1093/bioinformatics/btu638 25260700 PMC4287950

[B4] Andrews S. (2010) FastQC: A Quality Control Tool for High Throughput Sequence Data. Available at: http://www.bioinformatics.babraham.ac.uk/projects/fastqc.

[B5] Barbeito-Andrés J. Pezzuto P. Higa L. M. Dias A. A. Vasconcelos J. M. Santos T. M. P.. (2020). Congenital Zika Syndrome is Associated With Maternal Protein Malnutrition. Sci. Adv. 6, eaaw6284. 10.1126/sciadv.aaw6284 31950075 PMC6954064

[B6] Bayless N. L. Greenberg R. S. Swigut T. Wysocka J. Blish C. A. (2016). Zika Virus Infection Induces Cranial Neural Crest Cells to Produce Cytokines at Levels Detrimental for Neurogenesis. Cell Host Microbe 20, 423–428. 10.1016/j.chom.2016.09.006 27693308 PMC5113290

[B7] Bhagat R. Prajapati B. Narwal S. Agnihotri N. Adlakha Y. K. Sen J.. (2018). Zika Virus E Protein Alters the Properties of Human Fetal Neural Stem Cells by Modulating microRNA Circuitry. Cell Death Differ. 25, 1837–1854. 10.1038/s41418-018-0163-y 30050059 PMC6180120

[B8] Bond G. L. Hu W. Bond E. E. Robins H. Lutzker S. G. Arva N. C.. (2004). A Single Nucleotide Polymorphism in the MDM2 Promoter Attenuates the p53 Tumor Suppressor Pathway and Accelerates Tumor Formation in Humans. Cell 119, 591–602. 10.1016/j.cell.2004.11.022 15550242

[B9] Caires-Júnior L. C. Goulart E. Melo U. S. Araujo B. H. S. Alvizi L. Soares-Schanoski A.. (2018). Discordant Congenital Zika Syndrome Twins Show Differential In Vitro Viral Susceptibility of Neural Progenitor Cells. Nat. Commun. 9, 475. 10.1038/s41467-017-02790-9 29396410 PMC5797251

[B10] Candelo E. Cochard L. Caicedo-Herrera G. Granados A. M. Gomez J. F. Díaz-Ordoñez L.. (2019). Syndromic Progressive Neurodegenerative Disease of Infancy Caused by Novel Variants in HIBCH: Report of Two Cases in Colombia. Intractable Rare Dis. Res. 8, 187–193. 10.5582/irdr.2019.01014 31523596 PMC6743429

[B11] del Campo M. Feitosa I. M. L. Ribeiro E. M. Horovitz D. D. G. Pessoa A. L. S. França G. V. A.. (2017). The Phenotypic Spectrum of Congenital Zika Syndrome. Am. J. Med. Genet. A 173, 841–857. 10.1002/ajmg.a.38170 28328129

[B12] Dumont P. Leu J. I. J. Della Pietra A. C. George D. L. Murphy M. (2003). The Codon 72 Polymorphic Variants of p53 Have Markedly Different Apoptotic Potential. Nat. Genet. 33, 357–365. 10.1038/ng1093 12567188

[B13] El Ghouzzi V. Bianchi F. T. Molineris I. Mounce B. C. Berto G. E. Rak M.. (2016). ZIKA Virus Elicits P53 Activation and Genotoxic Stress in Human Neural Progenitors Similar to Mutations Involved in Severe Forms of Genetic Microcephaly and P53. Cell Death Dis. 7, e2440. 10.1038/cddis.2016.266 27787521 PMC5133962

[B14] França G. V. A. Schuler-Faccini L. Oliveira W. K. Henriques C. M. P. Carmo E. H. Pedi V. D.. (2016). Congenital Zika Virus Syndrome in Brazil: A Case Series of the First 1501 Livebirths With Complete Investigation. Lancet 388, 891–897. 10.1016/S0140-6736(16)30902-3 27372398

[B15] Gomes J. A. Sgarioni E. Boquett J. A. Terças-Trettel A. C. P. da Silva J. H. Ribeiro B. F. R.. (2021). Association Between Genetic Variants In NOS2 And TNF Genes With Congenital Zika Syndrome and Severe Microcephaly. Viruses 13, 325. 10.3390/v13020325 33672623 PMC7924177

[B16] Hermeking H. (2012). MicroRNAs in the p53 Network: Micromanagement of Tumour Suppression. Nat. Rev. Cancer 12, 613–626. 10.1038/nrc3318 22898542

[B17] Id Said B. Malkin D. (2015). A Functional Variant in miR-605 Modifies the Age of Onset in Li-Fraumeni Syndrome. Cancer Genet. 208, 47–51. 10.1016/j.cancergen.2014.12.003 25683625

[B18] Kehdy F. S. Gouveia M. H. Machado M. Magalhães W. C. Horimoto A. R. Horta B. L.. (2015). Origin and Dynamics of Admixture in Brazilians and its Effect on the Pattern of Deleterious Mutations. Proc. Natl. Acad. Sci. U. S. A. 112, 8696–8701. 10.1073/pnas.1504447112 26124090 PMC4507185

[B19] Langmead B. (2010). Aligning Short Sequencing Reads With Bowtie. Curr. Protoc. Bioinformatics Chapter 11, Unit 11.7. 10.1002/0471250953.bi1107s32 PMC3010897 21154709

[B20] Langmead B. Salzberg S. L. (2012). Fast Gapped-Read Alignment With Bowtie 2. Nat Methods 9, 357–359. 10.1038/nmeth.1923 22388286 PMC3322381

[B21] Liu G. Chen X. (2006). Regulation of the p53 Transcriptional Activity. J. Cell. Biochem. 97, 448–458. 10.1002/jcb.20700 16288459

[B22] Liu L. Chen Z. Zhang X. Li S. Hui Y. Feng H.. (2019). Protection of ZIKV Infection-Induced Neuropathy by Abrogation of Acute Antiviral Response in Human Neural Progenitors. Cell Death Differ. 26, 2607–2621. 10.1038/s41418-019-0324-7 30952992 PMC7224299

[B23] Liu J. Yu H. Hu W. (2015). LIF Is a New p53 Negative Regulator. J. Nat. Sci. 1, e131. PMC4493903 26161442

[B24] Mochida G. H. (2009). Genetics and Biology of Microcephaly and Lissencephaly. Semin. Pediatr. Neurol. 16, 120–126. 10.1016/j.spen.2009.07.001 19778709 PMC3565221

[B25] Moudi M. Sargazi S. Heidari Nia M. Saravani R. Shirvaliloo M. Shakiba M. (2020). Polymorphism in the 3′-UTR of LIF But Not in the ATF6B Gene Associates With Schizophrenia Susceptibility: A Case-Control Study and In Silico Analyses. J. Mol. Neurosci. 70, 2093–2101. 10.1007/s12031-020-01616-6 32504404

[B26] Nithiyanantham S. F. Badawi A. (2019). Maternal Infection With Zika Virus and Prevalence of Congenital Disorders in Infants: Systematic Review and Meta-Analysis. Can. J. Public Heal. 110, 638–648. 10.17269/s41997-019-00215-2 PMC6964464 31077071

[B27] Pim D. Banks L. (2004). p53 Polymorphic Variants at Codon 72 Exert Different Effects on Cell Cycle Progression. Int. J. Cancer 108, 196–199. 10.1002/ijc.11548 14639602

[B28] Ritchie M. E. Phipson B. Wu D. Hu Y. Law C. W. Shi W.. (2015). Limma Powers Differential Expression Analyses for RNA-Sequencing and Microarray Studies. Nucleic Acids Res. 43, e47. 10.1093/nar/gkv007 25605792 PMC4402510

[B29] Santos C. N. O. Ribeiro D. R. Cardoso Alves J. Cazzaniga R. A. Magalhães L. S. de Souza M. S. F.. (2019). Association Between Zika Virus Microcephaly in Newborns With the Rs3775291 Variant in Toll-Like Receptor 3 and Rs1799964 Variant at Tumor Necrosis Factor-α Gene. J. Infect. Dis. 220, 1797–1801. 10.1093/infdis/jiz392 31352487

[B30] Tang H. Hammack C. Ogden S. C. Wen Z. Qian X. Li Y.. (2016). Zika Virus Infects Human Cortical Neural Progenitors and Attenuates Their Growth. Cell Stem Cell 18, 587–590. 10.1016/j.stem.2016.02.016 26952870 PMC5299540

[B31] Teng Y. Liu S. Guo X. Liu S. Jin Y. He T.. (2017). An Integrative Analysis Reveals a Central Role of P53 Activation Via MDM2 in Zika Virus Infection Induced Cell Death. Front. Cell. Infect. Microbiol. 7, 327. 10.3389/fcimb.2017.00327 28775961 PMC5517408

[B32] Xiao J. Lin H. Luo X. Luo X. Wang Z. (2011). MiR-605 Joins p53 Network to Form a P53:miR-605:Mdm2 Positive Feedback Loop in Response to Stress. EMBO J. 30, 524–532. 10.1038/emboj.2010.347 21217645 PMC3034018

[B33] Zhang F. Hammack C. Ogden S. C. Cheng Y. Lee E. M. Wen Z.. (2016). Molecular Signatures Associated With ZIKV Exposure in Human Cortical Neural Progenitors. Nucleic Acids Res. 44, 8610–8620. 10.1093/nar/gkw765 27580721 PMC5063002

